# A Comparison of Oral and Intravenous Mouse Models of Listeriosis

**DOI:** 10.3390/pathogens7010013

**Published:** 2018-01-20

**Authors:** Michelle G. Pitts, Sarah E. F. D’Orazio

**Affiliations:** Department of Microbiology, Immunology & Molecular Genetics, University of Kentucky, 800 Rose Street–MS417, Lexington, KY 40536-0298, USA; michelle.pitts@uky.edu

**Keywords:** *Listeria monocytogenes*, intracellular bacteria, foodborne pathogen, host susceptibility, virulence

## Abstract

*Listeria monocytogenes* is one of several enteric microbes that is acquired orally, invades the gastric mucosa, and then disseminates to peripheral tissues to cause systemic disease in humans. Intravenous (i.v.) inoculation of mice with *L. monocytogenes* has been the most widely-used small animal model of listeriosis over the past few decades. The infection is highly reproducible and has been invaluable in deciphering mechanisms of adaptive immunity in vivo, particularly CD8^+^ T cell responses to intracellular pathogens. However, the i.v. model completely bypasses the gut phase of the infection. Recent advances in generating both humanized mice and murinized bacteria, as well as the development of a foodborne route of transmission has reignited interest in studying oral models of listeriosis. In this review, we analyze previously published reports to highlight both the similarities and differences in tissue colonization and host response to infection using either oral or i.v. inoculation.

## 1. *Listeria monocytogenes* Cause Foodborne Disease in Humans

*Listeria monocytogenes* are Gram-positive bacteria that can readily adapt to growth in a wide variety of environments ranging from soil to refrigerated deli meat to the cytosol of mammalian cells [1]. Human disease results when ready-to-eat food products are contaminated with *L. monocytogenes* during processing and then ingested without being heated [2,3,4]. Listeriosis ranges in severity from mild, self-limiting gastroenteritis to life-threatening bacteremia and meningoencephalitis. The high attack rate for gastrointestinal symptoms and the relatively low incidence of systemic diseases [5,6] strongly suggest that there are anatomic and/or innate immune bottlenecks in the gut that influence the severity of disease in humans.

Listeriosis was originally studied in rabbits, and the species was named monocytogenes due to the massive monocytosis observed in rabbit blood [7]. However, mouse models of infection were quickly developed in the 1980s after it was discovered that *L. monocytogenes* were the cause of outbreaks of foodborne disease in humans. Early studies that attempted oral transmission concluded that rodents were relatively resistant to oral infection [8,9]. Thus, the small animal model most widely used over the past few decades has been intravenous (i.v.) or sometimes intraperitoneal (i.p.) inoculation of mice. This route of transmission is meant to mimic the more severe, life-threatening forms of systemic listeriosis, but it completely bypasses the initial gut phase of infection. Advances in generating mice that more closely resemble humans [10], as well as mouse-adapted strains of *L. monocytogenes* [11,12], have led to increased use of oral infection models in recent years. In this review, we summarize what is currently known about how the transmission route affects both the pathogenicity of *L. monocytogenes* and the host immune response to listeriosis. 

## 2. Parameters that Affect Mouse Models of Listeriosis

**Figure 1:**
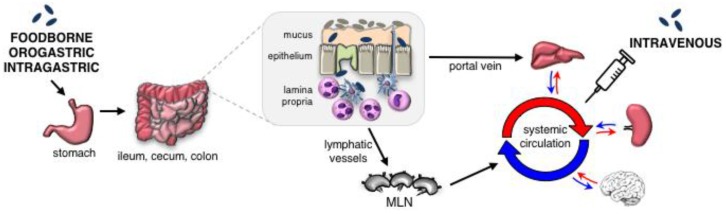
Orally-acquired *L. monocytogenes* must pass through multiple bottlenecks to reach the spleen or liver. Up to 90% of an ingested *L. monocytogenes* inoculum is either killed in the stomach or shed in feces within a few hours [16,18]. *L. monocytogenes* that survive must compete with the gut microbiota to gain access to the epithelium [19,20]; it has been estimated that invasion of the mucosal barrier is a rare event, with only 1 in 10^6^ bacteria reaching the underlying lamina propria [21]. If i.g. inoculation is used, or if an overwhelmingly large dose (≥10^9^ CFU) is used with any oral infection method, rapid dissemination to the liver, presumably via the portal vein, is observed. Otherwise, *L. monocytogenes* must disseminate to the mesenteric lymph nodes (MLN), avoid being killed by activated phagocytes, and then gain access to the blood circulation. Given the number of barriers faced, it is likely that small numbers of *L. monocytogenes* reach the spleen and liver asynchronously. In contrast, *L. monocytogenes* that are i.v. injected seed the spleen and liver as a large bolus within 10–15 min after administration [22,23,24].

One reason the i.v. model is widely used is because it is highly reproducible. Mice given a lethal dose by the i.v. route generally succumb to the infection within four days, and sub-lethal infection results in CFU (colony forming units) in the spleen and liver that show little mouse-to-mouse variation [13,14,15]. Similar results have been observed by many different laboratories around the globe, which means that it is very easy to make comparisons between studies. In contrast, following oral transmission, it is common to see CFU counts in the intestines, spleen, or liver that differ by up to 100,000-fold amongst mice in the same sample group [11,16,17]. Some of this variation is likely due to the fact that orally-acquired bacteria must pass through a series of bottlenecks in order to reach peripheral tissues, such as the spleen or liver (Figure 1). For example, *L. monocytogenes* must survive passage through the harsh acidic conditions of the stomach, effectively compete with gut microbiota in the intestinal lumen, translocate across the gut mucosa, and avoid being killed by phagocytes in the underlying lamina propria in order to spread systemically. However, technological advances in various parameters of the mouse model have greatly improved the reproducibility of oral infection in mice in recent years. 

### 2.1. Transmission Route 

For i.v. infection, bacteria are injected directly into the lateral tail vein. This technique can be difficult to learn, but once mastered, does not typically cause variation between investigators. All of the inoculum enters the bloodstream as a bolus and is rapidly cleared from the circulation with nearly all of the bacteria deposited in either the spleen or liver within 10–15 min [22,23,24]. Retro-orbital injection of the venous sinus can also be used, but this procedure requires that mice be anesthetized [25], and many forms of murine anesthesia can alter the host immune response and increase susceptibility to infection [26,27]. Interestingly, Czuprynski et al. showed that use of sodium pentobarbital specifically lowered the resistance of orally-infected, but not i.v-infected, mice [28]. 

Successful delivery of an oral inoculum can pose a more daunting challenge [29]. Bacteria can be added to the drinking water, but it is difficult to control exactly how much water each mouse drinks. Intragastric (i.g.) inoculation with a feeding needle can ensure that all of the inoculum reaches the stomach, but this procedure can lead to esophageal trauma in a user-dependent manner [30]. Use of a thin, flexible catheter for orogastric inoculation may alleviate this difficulty. However, the most physiologically relevant means of oral transmission is via contaminated food. Bou Ghanem et al. showed that mice would readily pick up a piece of *L. monocytogenes*-contaminated bread and eat it on their own without the need for any type of restraint [31,32]. Housing the mice on raised wiring flooring to prevent coprophagy, and thus, avoiding any unintended re-inoculation by in the ingestion of *L. monocytogenes* shed in feces, greatly improved the reproducibility of subsequent CFU burdens in infected tissues [16].

### 2.2. Mouse Strains 

The inbred mouse strains most commonly used to study systemic listeriosis are BALB/c mice (which display the highest level of susceptibility) and C57BL/6 or C57BL/10 mice, which are more resistant [13,33,34]. Other laboratory strains, such as C3H/He, CBA/J, and A/J mice, have intermediate phenotypes. The same general pattern of susceptibility and resistance has been observed using oral infection models [16,35,36]. An early study by Cheers and McKenzie found no difference in survival curves for male or female animals infected intravenously [13]. However, Pasche et al. observed that female mice were more susceptible to i.v. infection, regardless of the mouse strain used, with females having both higher CFU and decreased survival compared to males [37]. In agreement with that study, BALB/c females were more susceptible than BALB/c male mice following foodborne transmission [16]. However, male and female C57BL/6 mice had similar bacterial burdens in the ileum, colon, spleen, liver, gall bladder, and brain, suggesting that there was no gender-dependent phenotype in this mouse strain. Both studies used the *L. monocytogenes* EGDe strain, but the mice were purchased from different sources, they were used at two different age ranges (6–9 weeks vs. 12–14 weeks), and the mice were infected at different times of the day. Thus, further studies will be needed to determine whether there are gender-dependent differences that can be observed using only i.v. or oral infection models. 

Another reason that the i.v. model was favored over the past few decades was the belief that mice were largely resistant to oral infection due to the species specificity of the interaction between the bacterial surface protein internalin A (InlA) and E-cadherin expressed on the basolateral surface of intestinal epithelial cells. InlA binds with high affinity to E-cadherin and mediates a “zipper” type mechanism of bacterial uptake in human epithelial cells, but a single base pair change in murine E-cadherin restricts this interaction [38]. Two different humanized mouse lines were created to overcome this species barrier: a transgenic mouse that expresses human E-cadherin selectively in enterocytes of the small intestine [39] and a knock-in mouse with an E16P mutation that allows for InlA-mediated uptake in all E-cadherin-expressing cells [40]. Use of a humanized mouse increases InlA-mediated invasion in the small intestine by about 10-fold and in the large intestine by up to 100-fold [39,40]. However, *L. monocytogenes* that lack *inlA* can readily establish infection in the gut in wild-type mice [16], indicating that it is not necessary to modulate the species barrier to promote invasion of the intestinal epithelium.

### 2.3. Bacterial Strains 

A variety of different *L. monocytogenes* isolates have been used to study both i.v. and oral infection; neither model is restricted to the use of a particular strain. Wollert et al. developed a mouse-adapted derivative of *L. monocytogenes* EGDe with a modified version of InlA (InlA^m^) that binds murine E-cadherin with the same affinity as for the native InlA: human E-cadherin interaction [11]. Other groups have generated similar InlA modifications or moved the original InlA^m^ construct into other *L. monocytogenes* strains [12,41,42]. In all cases, use of the mouse-adapted strains results in about 10-fold greater CFU in mouse tissues, but expression of the InlA^m^ protein is not required to establish intestinal infection. Tsai et al. showed that the modifications of InlA^m^ that allowed for high affinity binding to E-cadherin also promoted binding to N-cadherin, and this resulted in enhanced uptake by villous M cells in the gut [43]. In that study, in which 10^10^ CFU were inoculated (i.g.) into either wildtype or E16P knock-in C57BL/6 mice, the InlA^m^-expressing strain caused greater neutrophil influx and compromised barrier function as assessed by microscopic analysis of the villus tips in the ileum. These results suggested that the altered tropism for intestinal invasion could lead to enhanced inflammation in the gut. However, Jones et al. used a flow cytometric approach to show that foodborne infection of BALB/c mice with slightly lower doses of either wild-type or InlA^m^-expressing bacteria resulted in similar inflammatory infiltrates in the large intestine [44]. More studies are clearly needed to resolve this issue, but investigators that use InlA^m^-expressing strains should be aware of these differences. 

The most commonly used strains of *L. monocytogenes* are 10403s, EGD, and EGDe; all three have been used extensively for in vivo studies in mice [45]. However, an exclusive focus on these strains may cause investigators to miss important facets of pathogenesis. For example, Czuprynski et al. found that two clinical isolates (Scott A and 101M) were more virulent than strain EGD following i.g. infection [46]. In a much larger analysis of over 6000 isolates collected in France, Maury et al. found that strains belonging multi-locus sequence type clonal complex CC1, CC4, CC6 reached significantly higher titers in either the liver or the brain following i.g. infection of E16P knock-in mice [47]. In another example, Quereda et al. showed that strain F2365, originally isolated during a 1985 California outbreak, was significantly more efficient in establishing oral infection than either EGDe or 10403s [48]. The enhanced virulence of strain F2365 was attributed to production of listeriolysin S (*llsA*), a bacteriocin that targets microbiota such as *Alloprevotella*, *Streptococcus, Allobaculum ,*and *Lactococcus* species [20,49]. Indeed, gnotobiotic mice mono-associated with various *Lactobacillus* species were much less susceptible to oral *L. monocytogenes* challenge [50,51,52], which suggests that *Lactobacillus* species compete with *Listeria* for survival in the gut lumen. An *llsA* deletion mutant had a significant colonization defect in both the gut and in peripheral tissues when mice were infected orally, but there was no difference in CFU burdens in the spleen or liver following i.v. infection [20,48]. These results highlight the fact that *L. monocytogenes* growth and survival in the spleen and liver can vary depending on whether the bacteria seed these tissues directly from a bloodstream injection or via dissemination from an active infection in the gut. 

### 2.4. Infectious Dose 

A key difference between i.v. and oral models of listeriosis is the dose required to establish infection. For most *L. monocytogenes* strains, the LD_50_ for i.v. infection is approximately 10^4^ CFU for highly susceptible BALB/c mice and 10^5^ to 10^6^ CFU for more resistant mouse strains [13]. Just a few hundred CFU are sufficient to establish an infection that induces protective immunity against subsequent challenges with up to ten-fold more than the lethal dose. In contrast, extremely high doses (10^9^–10^11^ CFU) were used for oral infection in most early studies; this likely reflected the belief that mice were resistant to oral infection and was an attempt to combat the reproducibility issues associated with oral infection studies at the time. Doses in this range are technically challenging to work with as it is difficult to suspend the large biomass of that many bacteria in the small volumes typically needed for administration in mice. More recent work using foodborne transmission demonstrated that intestinal colonization could be established with doses as low as 10^7^ CFU in susceptible BALB/c mice [16,32]. Bou Ghanem et al. reported that careful attention to several parameters of the infection model led to increases in reproducibility for CFU burdens which obviated the need to use an overwhelmingly high inoculum. These included the use of young (6–8 weeks old) mice, suspending the inoculum in a liquid with a high fat content (butter), and housing the mice on raised wire flooring to prevent coprophagy [16,31,32]. 

Even with these improvements to the foodborne model, oral infection still requires inocula that are at least 1000-fold higher than used for i.v. infection. This greatly limits the range of doses that can be used for any particular experiment, and means that lethality can be achieved only in highly susceptible mouse strains like BALB/c. Based on in vitro survival tests and the number of CFU shed in feces within hours of oral administration, it is presumed that up to 90% of any ingested bacteria are killed in the stomach. However, *L. monocytogenes* that do survive in the intestinal lumen are gut-adapted, and display σB-dependent phenotypic changes that promote survival at low pH and invasion of epithelial cells [53,54]. For the first 24 to 36 h after foodborne transmission, *L. monocytogenes* are detected only in the intestinal tissues and the draining mesenteric lymph nodes; dissemination to the spleen and liver consistently occurs by 48 hpi [16]. In contrast, with i.g. inoculation some investigators find rapid spread to the spleen and liver within just 4 h [11,36,39,55] while others saw no dissemination until 48 h post-infection [9,12,56]. These observations suggest that there is a user-dependent mechanism that can result in rapid dissemination of orally acquired *L. monocytogenes.* Such a mechanism would bypass the gut colonization stage, and the physiological relevance of this pathway to human disease is not yet clear. If a large number of bacteria rapidly reach the bloodstream due to physical trauma, then the mice have essentially been infected by the i.v. route even though the published report characterizes the infection as orally acquired. As such, it can be difficult to evaluate infections initiated by i.g. injection unless the study included an early time point (≤24 h post-infection) to assess CFU burdens in the spleen and liver. 

### 2.5. Microbiota

Although pre-treatment with antibiotics to reduce the density of gut microbiota is a common feature of other mouse models of enteric infection, it has not been frequently used to study listeriosis. Early studies did show that gnotobiotic rodents were significantly more susceptible to oral challenge than conventionally raised animals [57,58,59]. However, methods to reduce colonization resistance were not actively pursued until recently, perhaps due to the wide popularity of the i.v. model, and the fact that intestinal infection could be established in mice without disrupting the gut microbiota. Becattini et al. demonstrated that oral infection in mice could be achieved with doses as low as 10^2^ CFU if the animals were pre-treated with either streptomycin, clindamycin, or a cocktail of four antibiotics [19]. Their data suggest that specific bacteria within the order Clostridiales inhibit the growth and survival of *L. monocytogenes* in the intestinal lumen. These findings may help to explain why the incidence of listeriosis is relatively low in humans even though the frequency of detecting contaminated food products is quite high and has been increasing in recent years.

The effects of the microbiome on systemic listeriosis have not yet been clearly elucidated. Two groups showed that i.v. infection of germfree mice resulted in a 10-fold increase in CFU in the spleen and liver [60,61]. Since the inoculum bypassed the gut in these studies, the mechanism of increased susceptibility was not reduced colonization resistance due to a lack of microbiota. Indeed, in one study, no difference in CFU burdens in the spleen or liver was noted if the mice were pre-treated with antibiotics prior to i.v. infection [60]. A more likely explanation is that the density and composition of the gut microbiota influences both the development of innate immunity and specific immune responses. For example, dos Santos et al. showed that splenocytes from mice mono-associated with *Lactobacillus* and then infected with *L. monocytogenes* (i.p.) produced more TNFα and IFNγ than spleen cells from germfree mice [52]. 

## 3. Pathogenesis of *L. monocytogenes* Infection

Unfortunately, very few studies have directly compared i.v. and oral transmission routes to study *L. monocytogenes* virulence factors that enhance survival in the mammalian host. In general, investigators tend to use the i.v. model for a quick and easy readout in the spleen or liver, and only use oral inoculation when the gut is the focus of the study. The factors needed to promote survival of *L. monocytogenes* in the intestinal lumen are reviewed elsewhere in this issue [Becattini and Pamer, Pathogens]; here we will focus on similarities and differences in systemic infection following either i.v. or oral transmission. The goal of such an analysis is to determine whether the early presence of *L. monocytogenes* in gut tissues affects the subsequent colonization of peripheral tissues such as spleen and liver. No bacteria are detected in the gut within 24–48 h of i.v. infection; however, once *L. monocytogenes* colonize the gall bladder, they will be spread to the gut each time ingested food induces the secretion of bile into the small intestine [60,62]. Direct comparison of the oral and i.v. transmission routes is further complicated by the timing of initial dissemination to peripheral tissues. *L. monocytogenes* administered intravenously reach the liver and spleen simultaneously within 15 min of injection, but orally-acquired bacteria can take up to 48 h to reach these organs and since they pass through several bottlenecks, are likely to appear in asynchronous waves. 

### 3.1. Virulence Factors that Promote Survival in the Host 

A few of the classically studied *L. monocytogenes* virulence factors have been examined using both oral and i.v. infection models. Listeriolysin O (LLO) is a pore-forming toxin that facilitates vacuolar escape. Mutant strains that do not express LLO are severely attenuated during i.v. infection [63,64,65]. Likewise, Roll et al. showed that LLO was also needed for extraintestinal dissemination of *L. monocytogenes* in i.g.-inoculated mice (Roll, JT 1990). In that study, very few wild-type *L. monocytogenes* spread to the spleen within 24 hpi, suggesting that the i.g. inoculation did not cause excessive esophageal trauma, so a comparison of these data truly reflects different routes of administration. More recently, Kline et al. identified hibernation promoting factor (HBF) as a novel protein required pathogenesis of *L. monocytogenes* infection [66]. In that study, HBF-deficient bacteria were less virulent regardless of whether the bacteria were administered by the foodborne or i.v. route. 

Mutants for several other virulence factors have been tested in both oral and systemic models, but in each of these cases, i.g. inoculation was the method used for oral transmission, and CFU burdens were not assessed at any early time points, so it is difficult to determine if the majority of the inoculum passed through the gut tissue or if a rapid hematogenous route of spread occurred. For example, the *L. monocytogenes* iap gene, which codes for a murein hydrolase, called p60, was required for full virulence following either i.v. [67] or i.g. [68] inoculation. Faith et al. showed that there were 3–4 logs fewer CFU in the spleen and liver 3 days after i.g. inoculation; interestingly, they found much less of a defect for the p60 mutant in colonizing the cecum. InlB binds to the mammalian Met receptor and promotes uptake in some cell types such as hepatocytes. Infection with an inlB deletion mutant resulted in a defect in liver colonization regardless of whether the bacteria were administered by the i.v. or i.g. route [69,70,71]. *sigB* encodes an alternative sigma factor that is important for regulating expression of genes in Gram-positive bacteria during stress. Using a competition assay, Nadon et al. found only modest differences in recovering *sigB* mutant *Listeria* and wild-type *Listeria* from the spleen and liver of infected mice and there was no difference between the i.g-infected and i.p-infected animals [72]. 

### 3.2. Dissemination beyond the Spleen and Liver

In both i.v. and oral models of listeriosis, the spleen and liver are the first tissues infected during the systemic phase of the infection. After a period of time during which exponential replication occurs in these tissues, *L. monocytogenes* gain access to the circulatory system, and dissemination to other tissues occurs. The gall bladder appears to be a reservoir for extracellular replication of *L. monocytogenes* and is colonized late in the infection (three days after i.v. infection [18] and five days after foodborne infection [16]) regardless of the transmission route. Within 24 h after i.v. infection, large numbers of *L. monocytogenes* bacteria can be found in the bone marrow [73,74], but no bacteria were detected in the bone marrow of orally infected mice [73,75]. This difference could alter the activation status of hematopoietic cells in the marrow, particularly phagocytes of the myeloid lineage. Finally, dissemination to the brain is thought to be a late stage event in humans with an incubation period of weeks or months between the ingestion of contaminated food and symptoms of meningoencephalitis [76,77]. Bou Ghanem et al. showed that *L. monocytogenes* could be detected in the brains of susceptible BALB/c mice five days after ingestion of contaminated food [16]. In an earlier study, Altimira et al. found that repeated doses of *L. monocytogenes* resulted in brain lesions in some CD1 mice 7–10 days after oral administration [78]. In contrast, i.v. inoculation generally does not result in dissemination to the brain unless extremely high doses are used, resulting in high titer bacteremia within 48 h and rapid death of the animals [79]. Thus, the course of infection and the number of tissues involved varies depending on the route of transmission. 

## 4. Immune Responses to *Listeria monocytogenes*

The host immune response is the aspect of listeriosis that is most likely to differ depending on the route of transmission. When initial antigen encounter takes place at a mucosal site such as the intestinal epithelium, the types of immunoglobulin produced and the tissue localization of memory T cells differs compared to i.v. immunization. The mucosal immune system is designed to tolerate the presence of commensal microbes, so initiation of specific immunity to a pathogen like *L. monocytogenes* must overcome this hurdle. In addition, for modes of oral transmission that result in a discrete period of time during which *L. monocytogenes* colonize only the gut, the possibility exists that the infection will induce secretion of cytokines and chemokines that gain access to the systemic circulation. These inflammatory mediators could prime immune cells in distant sites such that phagocytes present in the spleen or liver are pre-activated and will no longer serve as an efficient replicative niche for *L. monocytogenes*.

After foodborne transmission of *L. monocytogenes*, there is a higher peak bacterial burden and delayed clearance in the liver compared with the spleen [16,41]. Although this trend is sometimes observed following i.v. infection [80], it is much more common to observe relatively equal bacterial loads in the spleen and liver [33,81,82,83,84,85]. It is reasonable to presume that the mechanisms used to clear infection in these two tissues would differ due to the nature of the cell types available for intracellular infection. Most of the bacterial load in the spleen is likely extracellular since lymphocytes, monocytes, and dendritic cells, which comprise more than 90% of splenocytes, do not serve as efficient intracellular niches for *L. monocytogenes* [44,75,86]. In the liver, *L. monocytogenes* multiply extensively and spread from cell-to-cell in hepatocytes [87], and are protected from extracellular innate immune clearance mechanisms. Thus, it is not surprising that there is a delay in bacterial clearance until later in the infection when cytolytic CD8^+^ T cells infiltrate the liver [88,89]. However, it is not yet clear why this differential clearance rate is not consistently observed following i.v. infection. 

### 4.1. Innate Immunity 

Early recognition of infection takes place when microbial associated molecular patterns (MAMP) bind to pattern recognition receptors, such as Toll-like receptors (TLR) and Nod-like receptors (NLR), that are expressed either on the cell surface or in specific compartments of the host cell. For example, Nod2 (also known as NLRC2) is an intracellular sensor that binds to the peptidoglycan derivative muramyl dipeptide. Kobayashi et al. showed that Nod2^-/-^ mice were significantly more susceptible to i.g. infection, but there was no difference in tissue burdens in mice infected by the i.v. or i.p. route [17]. Nod2 signaling was shown to be important for inducing a type I interferon (IFN) response, but only in situations where TLR signaling was suppressed, such as at the gut mucosa [90]. This could explain the lack of a phenotype in mice challenged systemically [17].

Cytosolic localization of *L. monocytogenes* also induces STING-dependent secretion of type I IFN, a process that has been well-studied in bone marrow-derived macrophages [91,92]. In vivo, mice on either the C57BL/6 or BALB/c background that lack the type I IFN receptor (IFNAR^-/-^) were significantly less susceptible to i.v. infection, which suggested that the type I IFN response was actually detrimental to the host [93,94,95]. Oral infection of IFNAR1^-/-^ mice gave conflicting results in two separate studies. Kernbauer et al. found increased *L. monocytogenes* LO28 in the liver after IFNAR-deficient C57BL/6N mice were i.g. infected [83]. Pitts et al. saw no difference in *L. monocytogenes* InlA^m^-expressing EGDe in the liver compared to wild-type BALB/c mice [96]. The differences in tissue burdens in the liver may to be due to rapid hematogenous spread of *L. monocytogenes* following i.g infection since Kernbauer et al. detected at least 10^4^ CFU within 24 h of injection. Robust production of IFNβ was detected in the spleens of wild-type mice after i.v. infection, but not after foodborne infection [35,96,97]. Thus, the downstream effects of type I IFN production differ considerably depending on the route of transmission.

Other aspects of innate immunity appear to be similar following either oral or i.v. infection. For example, Czuprynski et al. showed that neutrophils were important for clearing *L. monocytogenes* from the liver and spleen of mice infected intragastrically [98], confirming early studies performed with i.v. inoculation [99,100]. Ly6C^hi^ monocytes are another early responding cell type that infiltrates infectious foci within 24–48 h and this occurs following both i.v. and oral infection [75,101].

### 4.2. Adaptive Immunity

An effective T cell response is critical for achieving sterilizing immunity against listeriosis. In particular, cytotoxic CD8^+^ T cells are needed to lyse infected cells, releasing *L. monocytogenes* from their protected intracellular niche [102]. Dendritic cells are the most efficient antigen presenting cell type that can prime CD8^+^ T cells [103]. 

After i.v. infection, *L. monocytogenes* are filtered from the blood by either marginal zone macrophages or CD8^−^ dendritic cells. Current models suggest that shortly after that, CD8^−^ dendritic cells transport *L. monocytogenes* into the T cell zone of the spleen where they may undergo apoptosis and be engulfed by CD8^+^ dendritic cells that cross the present bacterial antigen to T cells [104,105,106]. Much less is known about how L. monocytogenes disseminate from the intestinal lamina propria to the draining MLN. *L. monocytogenes* associates with both CD103^+^ and CD103^−^ dendritic cells in the gut [44], so it is possible that these cells transport cell-associated bacteria to the T cell zone of the MLN in a manner analogous to that observed in the spleen.

Antigen-specific CD4 and CD8 T cells are known to be primed during either oral or i.v. infection [107], and memory T cells form that protect against secondary challenge using either infection model [108,109]. However, there are both qualitative and quantitative differences in the T cell responses that depend on the route of transmission. For example, Huleatt et al. found that i.g. infection of BALB/c x C57BL/6 F1 mice resulted in nearly five times more *Listeria*-specific T cells in the intestinal lamina propria compared to the spleen [110]. The TCR Vb chain usage varied for T cells found in the spleen compared to the gut, but there was no correlation with the route of transmission. In a more recent study, Sheridan et al. found that CD103^+^ memory T cells rapidly formed following foodborne infection and that the cells that homed to the intraepithelial region of the gut and became resident cells were critical for protection against subsequent challenge [109]. Foodborne infection also stimulates a robust γδ T cell response that is important for full protective immunity [111,112]. These non-circulating memory T cells were resident in the MLN and actively secreted IL-17A. γδ memory T cells have been found in the mediastinal lymph nodes of mice given i.p. injections of *Staphylococcus aureus* [113], so it is possible that these cells are also stimulated during systemic listeriosis, but that remains to be determined. 

## 5. Conclusions

Although there are, clearly, some similarities between the oral and i.v. models of listeriosis, the infections also differ in critical ways. Thus, it is worth re-examining the rationale for frequent use of the i.v. model. Listeriosis, like most human infections, does not begin with large numbers of bacteria gaining direct access to the bloodstream. It is possible that many of the early innate immune recognition events that occur in response to i.v. infection represent an artifact created when a large bolus of bacteria reaches the spleen all at once. Refinement of the oral infection model, including the development of targeted ways to increase the efficiency of competition with gut microbiota, will improve our understanding of how *L. monocytogenes* naturally disseminate from the gut to the spleen, liver, and brain. The biggest remaining challenge for the oral model seems to be the use of multiple inoculation methods by various investigators, which makes direct comparison of published studies more difficult. We would argue that foodborne transmission is physiologically relevant, is easy to learn, and is less prone to user-dependent variations. Future *L. monocytogenes* research would benefit from the use of an infection model that mimics the natural transmission as closely as possible.
